# BioUML: an integrated environment for systems biology and collaborative analysis of biomedical data

**DOI:** 10.1093/nar/gkz440

**Published:** 2019-05-27

**Authors:** Fedor Kolpakov, Ilya Akberdin, Timur Kashapov, llya Kiselev, Semyon Kolmykov, Yury Kondrakhin, Elena Kutumova, Nikita Mandrik, Sergey Pintus, Anna Ryabova, Ruslan Sharipov, Ivan Yevshin, Alexander Kel

**Affiliations:** 1 BIOSOFT.RU, LLC, Novosibirsk 630090, Russian Federation; 2 Institute of Computational Technologies SB RAS, Novosibirsk 630090, Russian Federation; 3 Novosibirsk State University, Novosibirsk 630090, Russian Federation; 4 Institute of Cytology and Genetics SB RAS, Novosibirsk 630090, Russian Federation; 5 geneXplain GmbH, 38302 Wolfenbüttel, Germany; 6 Institute of Chemical Biology and Fundamental Medicine, SB RAS, Novosibirsk 630090, Russian Federation

## Abstract

BioUML (homepage: http://www.biouml.org, main public server: https://ict.biouml.org) is a web-based integrated environment (platform) for systems biology and the analysis of biomedical data generated by omics technologies. The BioUML vision is to provide a computational platform to build virtual cell, virtual physiological human and virtual patient. BioUML spans a comprehensive range of capabilities, including access to biological databases, powerful tools for systems biology (visual modelling, simulation, parameters fitting and analyses), a genome browser, scripting (R, JavaScript) and a workflow engine. Due to integration with the Galaxy platform and R/Bioconductor, BioUML provides powerful possibilities for the analyses of omics data. The plug-in-based architecture allows the user to add new functionalities using plug-ins. To facilitate a user focus on a particular task or database, we have developed several predefined perspectives that display only those web interface elements that are needed for a specific task. To support collaborative work on scientific projects, there is a central authentication and authorization system (https://bio-store.org). The diagram editor enables several remote users to simultaneously edit diagrams.

## INTRODUCTION

The BioUML project was started in 2002, and its main goal was to develop a common purpose visual language for formal descriptions of the structure and function of biological systems—a Biological Universal Modelling Language (BioUML) (1). As a starting point, we used the graphic notation suggested by the GeneNet system (2,3). We have developed several diagram types that allow the user to construct a biological model step-by-step, with increasing levels of detail and formality. Through this approach, we have developed two databases: Biopath (4) and Cyclonet (5).

Subsequently, the international community created the Systems Biology Graphical Notation (SBGN), which standardizes the graphical notation used in maps of biological processes (6). The BioUML team was involved in this standardization process. Currently, BioUML completely supports the SBGN Process Description diagrams.

From the beginning, BioUML has supported a paradigm of visual modelling, where the user can create a diagram that completely and formally specifies the given biological model. BioUML then automatically generates program code that is used to simulate the model behaviour. The initial versions of the BioUML workbench generated MATLAB code and used the MATLAB ODE suite for simulation (7). The current version of BioUML generates highly optimized Java code and uses its own state-of-the-art simulation engines, which have been developed over the last 14 years. Thanks to the optimized simulation engines, BioUML is the only tool that can pass the SBML (Systems Biology Markup Language) semantic test suite (http://sbml.org/Facilities/Database/Submission/Details/108)—a comprehensive set of tests to verify the correctness of simulation engines for complex biological systems (8).

The paradigm of systems biology implies not only computational modelling but also the so-called ‘dry-wet-dry’ cycle composed of theory and computational modelling, which proposes specific testable hypotheses about a biological system, followed by experimental validation and further refinement of the computational model or theory using the newly acquired quantitative description of cells or cell processes (9). Since the objective of such modelling is to describe, as fully as possible, the entire set of interactions in a biological system, high-throughput systems and genome-wide experimental techniques (so-called ‘omics’ data—transcriptomics, metabolomics, proteomics, etc.) are most suitable for validation of such system models. Such high-throughput techniques are mainly used to collect quantitative data for the construction and validation of models. Thus, the second main direction of BioUML development was the processing and analysis of omics data.

To process and analyse omics and other biomedical data, we integrated R/Bioconductor (10) and Galaxy (11) into the BioUML platform and developed 300+ methods for data analysis. To support the concept of reproducible research, the BioUML platform is equipped with a workflow engine that helps to place various methods for analysis into consequent chains/pipelines that can repeatedly perform the same sequence of analytical steps with new input data. BioUML provides a powerful web editor to visually construct such workflows and an engine for the execution of workflows on a server or computer cloud.

For the visualization of omics data, the BioUML platform provides a diagram viewer/editor and a genome browser. Data from omics experiments (transcriptomics, proteomics, metabolomics) can be mapped on different biological pathways and visualized by highlighting corresponding nodes on the diagram.

Thus, BioUML can perform two steps of the system biology ‘dry-wet-dry’ cycle—the modelling and analysis of omics data. The main BioUML vision is to provide a computational platform for building virtual cells, virtual physiological humans and virtual patients.

Currently, scientists from different countries use the BioUML platform for the collaborative reproducible analysis of biomedical data, pathway visualization and genome browsing.

## ARCHITECTURE OVERVIEW

### Meta-model

The meta-model is the core of the BioUML platform. It provides an abstract layer (compartmentalized attributed graph) for the comprehensive formal description of a wide range of biological systems and other complex systems. The content of biological pathway databases (e.g. Reactome, PantherDB), SBML models, biological pathways in BioPAX format (12), as well as workflows, can be expressed in terms of the meta-model. This formal description can be used both for a visual representation of the structure of biological systems and for automated code generation to simulate the model behaviour.

The meta-model describes a system as three interconnected parts: (i) graph structure: the system structure is described as a compartmentalized graph; (ii) database level: each graph element can contain a reference to an object in biological databases; and (iii) mathematical (executable) model—any graph element can be associated with an element of a mathematical model or an analysis method (e.g. for workflows).

The meta-model structure is problem-domain neutral, so it is used to describe biological models, as well as executable workflows, for the analysis of biomedical data.

### Plug-in based architecture

BioUML is based on plug-in architecture (Open Services Gateway Initiative; OSGi) that enables extension of the platform functionality by the addition of new plug-ins. The basic components of the plug-in-based architecture are as follows:

- A plug-in is the smallest unit that can be developed and delivered separately in the BioUML platform. Plug-ins are coded in Java. A typical plug-in consists of Java code in a JAR library, several read-only files and other resources, such as images, message catalogues and native code libraries. A plug-in is described in an XML manifest file called plugin.xml. The parsed contents of plug-in manifest files are made available programmatically through a plug-in registry API provided by the Eclipse runtime.
- Extension points are well-defined function points in the system where other plug-ins can contribute functionality.
- An extension is a specific contribution to an extension point. Plug-ins can define their own extension points so that other plug-ins can integrate tightly with them.

Currently, the BioUML platform includes 120+ plug-ins (http://wiki.biouml.org/index.php/Category:Plugins) and 36 extension points (http://wiki.biouml.org/index.php/Category:Extension_points).

## USER INTERFACE

**Figure 1.**
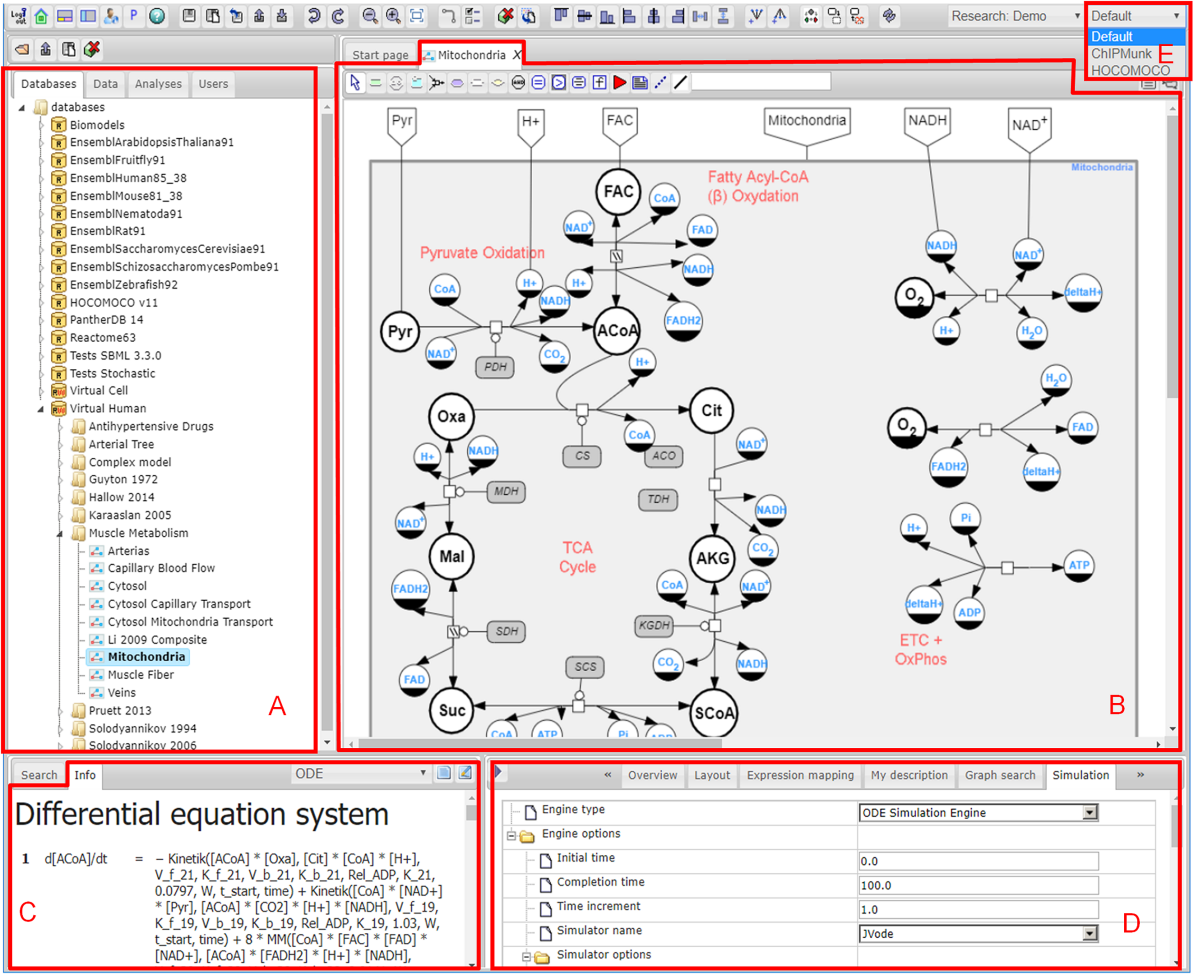
The BioUML web interface consists of: (**A**) repository pane, (**B**) document pane, (**C**) info pane, (**D**) viewer/editor pane, (**E**) perspective selector.

The BioUML user interface (Figure 1) and architecture were inspired by the Eclipse platform (https://www.eclipse.org/ide). The web interface of BioUML is implemented as a single page application and comprises the following main parts:

- **Repository pane**: This pane contains three tabs—database navigation, user data and available methods.
  - **Databases**: On the top level, this contains a list of available biological databases. Each database has its own structure. Usually, it consists of:
    - Data: Collections of biological objects (genes, proteins, chemical substances, reactions, etc.)
    - Diagrams: Diagrams or models of biological pathways.
  - **Data**: This contains user data organized into projects. The user can create their own project, import omics and other data, analyse the data and invite colleagues for collaborative analyses of corresponding data. All projects (their own and those where the user was invited) are shown in the ‘Collaboration’ folder. A number of projects were created to demonstrate the main possibilities of the BioUML platform.
  - **Analyses**: A set of analyses and workflows. The set is divided into four sections:
    - Galaxy: Methods of analyses that are available from the Galaxy platform installed on the same server or cloud.
    - Methods: Analysis methods implemented in Java within the BioUML platform. Currently, it contains 300+ methods grouped into 15 categories. Each method has a detailed description and its own page in the BioUML wiki (e.g. http://wiki.biouml.org/index.php/Cluster_analysis_by_K-means_(analysis)).
    - JavaScript: JavaScript API for access to analysis methods.
    - Workflows: Ready workflows for the analysis of omics data.
  - **Document pane**: This is the main working area in the BioUML user interface where tabs with individual documents are opened. There are several document types that provide different functionalities. The main document types are diagram (model), workflow, analysis method and genome browser.
  - **Viewer/editor pane**: Each document has a set of tabs (viewers and editors) to work with the current type of documents. Thus, a model document has the following tabs: (i) overview—a small diagram view for navigation; (ii) description; (iii) parameter; (iv) variables—used to display and edit the model parameters and values; (v) simulation—allowing the user to select and start a simulation engine and to configure the simulation parameters; (vi) plot—displays the simulation results; (vii) layout—used to select the graph layout algorithm and apply it to the diagram; (viii) expression mapping—to map and display omics data on the diagram.
  - **Info pane:** This shows a description of the selected object in the repository or in the diagram pane. These descriptions are generated using templates and can be opened in a separate tab in the web browser. The user can select a template if several templates are available. For example, for a diagram (model), the following templates are available: (i) reactions—a list of reactions from the model; (ii) parameters—a list of parameters and their values and measurement units; (iii) variables—a list of variables and their initial values and measurement units; (iv) ODE—a system of differential equations generated from the model; (v) overview—includes all of the information mentioned above.
  - **Search pane**: This allows the user to specify criteria for searching information in any database selected in the repository. For this purpose, information of the installed databases is indexed using Apache Lucene (http://lucene.apache.org).
  - **Perspectives**: This facilitates user concentration on a specific task or a specific database. We have developed several predefined perspectives that display only those web interface elements that are necessary for the specific task.

## MAIN FEATURES

### Systems biology

BioUML supports the main worldwide standards used in systems biology:

- **SBML:** Systems Biology Markup Language (13) serves for the formal description of mathematical models. BioUML supports all versions of SBML, from l1v2 to the latest l3v2, including the extension packages ‘fbс’ (14) and ‘comp’ (15).
- **SBGN:** Systems Biology Graphic Notation (6) is used for the visual description of model elements (complexes, compartments, molecule types, reactions, etc.). BioUML completely supports SBGN Process Description diagrams and uses them to visually represent SBML models. BioUML also supports the XML markup language SBGN-ML (https://github.com/sbgn/sbgn/wiki/SBGN_ML), which facilitates the exchange of SBGN diagrams between tools.
- **Antimony**: Human-readable text format that supports most of the SBML features (16). In BioUML, it is automatically processed into SBML diagrams in SBGN notation. BioUML supports import and export into the antimony format.
- **SedML**: Simulation Experiment Description Markup Language (17) describes model simulation steps and facilitates the reproducibility of simulation experiments. In BioUML, it is translated into workflows, which allows for the analysis and simulation of mathematical models and bioinformatics data.
- Many models, however, require some features that are missing from the above-mentioned standards. In these cases, the SBML standard provides extension mechanisms via the <notes> and <annotation> XML elements. Using these extensions, BioUML stores all additional information about the models (e.g. diagram view attributes and layout).
- SBGN was developed independently from SBML, so it does not define visual syntaxes for events, functions, assignments and other mathematical elements. To solve this problem, we have extended the SBGN process diagrams with additional glyphs to represent and use them in our own notations. Detailed information about the types of models and their visual representations can be found at http://wiki.biouml.org/index.php/Diagram_type.
- **Simulation engine**: BioUML automatically generates program code that is used to simulate the behaviour of the analysed model. Currently, BioUML generates highly optimized Java code and uses its own state-of-the-art simulation engines. For each diagram, it provides a list of available engines. For example, network of reactions can be simulated as a system of ODEs or as a Gillespie-type stochastic model. The selected simulation engine provides a list of available solvers. Available ODE solvers include JVODE, which is a package CVODE ported from C to Java and developed at the Lawrence Livermore National Laboratory (18). It utilizes the multistep Adams-Moulton method and the backward differential algorithm, RADAU5 solver (19), as well as classic algorithms (Euler, Dormand-Prince (20)). The stochastic simulation engine provides the exact methods, Gillespie (21) and Gibson-Bruck (22), as well as approximation methods.

Diagram transformation into a simulatable state by the selected simulation engine is a prerequisite for simulation. Thus, a hierarchical diagram may be transformed to an ordinary ‘flat’ diagram with reactions and entities. An agent-based diagram may be partially flattened, where all subdiagrams of the same type may be transformed into one combined agent.

There are several other simpler preprocessors. For example, SBML constraints are transformed into discrete events, thereby halting simulation when the constraint is violated. Additionally, fast reactions are transformed into algebraic equations, and Boolean expressions are transformed into numeric expressions, etc.

Other simulation engines are:

- Hemodynamics: specifically tailored to solve PDE problems describing blood flow in arteries.
- Population: solves NLME problems using the R library.
- Dynamic FBA: dynamically runs Flux Balance Analysis simultaneously with ODE simulation.

### Modular modelling

In a modular approach, the investigated system is viewed as a set of interconnected subsystems. Each subsystem can be considered and simulated independently. Integration of these models (or modules) results in a more complex model of the whole system. Modules may leverage different mathematical formalisms and scales. They can be created, validated and improved independently and may be viewed as replaceable parts. Modules provide explicit interfaces through which they can be connected without exposing their inner structure to the user. We consider modules as mathematical models; their interfaces are variables and constant parameters. For example, the value of a variable in one model may be constant, while in another model it changes dynamically. Numerical calculations are performed in two ways:

- Flattening: A modular model may be transformed into a non-modular model by aggregating all elements of all modules with automatic resolving of established connections between variables (23).
- Agent-based simulation: Each module is simulated independently with its own simulator and formalism. The scheduler coordinates their interactions by sending and receiving numerical values of the connected variables (24).

### Parameter estimation

BioUML provides several stochastic and deterministic global optimization methods (25), including a stochastic ranking evolution strategy (26), particle swarm optimization (27), cellular genetic algorithms (28) and others. We have achieved a significant acceleration of these methods using concurrent computing. Algorithms can use experimental data in time-course or steady state forms, with exact or relative values. BioUML also supports multi-experiment parameter estimation. A detailed comparison with other software can be found in (25).

### Model analysis

We have implemented a number of methods for model analysis and reduction, including:

- Identifiability analysis infers how well the model parameters are approximated by the amount and quality of experimental data (29,30).
- Search for linear, monomolecular and pseudo-monomolecular reactions (31).
- Quasi-steady state analysis (32).
- Sensitivity analysis of the model steady state (33).
- Metabolic control analysis quantifies how fluxes and species concentrations depend on the system parameters (34).
- Stoichiometric analysis derives linear relationships between flux rates and reactant concentration derivatives (31).
- Mass conservation analysis decomposes a stoichiometric matrix into the product of its linearly independent rows and a link matrix (35).

## BIOMEDICAL DATA ANALYSES

For processing and analysis of omics and other biomedical data, we have integrated the best platforms in the respective fields—R/Bioconductor (36) and Galaxy—into the BioUML platform and developed 300+ of our own analysis methods (http://wiki.biouml.org/index.php/Category:Analyses).

- **Integration with R**. BioUML has bidirectional integration with R. R scripts can be used within BioUML in four ways: (i) The user can create, edit and execute R scripts in the BioUML document pane. The editor supports syntax highlighting; (ii) The ‘Script’ pane allows the user to input and execute R commands; (iii) R scripts can be building blocks of a BioUML workflow; and (iv) There are a number of Java analysis tools that provide a convenient interface to configure the analysis parameters, with subsequent generation of the corresponding R script. To execute an R script, the BioUML server calls R. Text output is shown in the ‘Output’ tab. Graphical results (plots, dendrograms, etc.) are shown on separate pages.

To gain access to the BioUML server from inside R, we have developed the rbiouml package (https://cran.r-project.org/package=rbiouml). The package contains functions to acquire data from the BioUML repository, import/export the data, start analyses and workflows and manage the execution queue.

- **JavaScript API**. The user can use JavaScript (document, console, building block on workflow) similar to R scripts. API provides functions to acquire data from the BioUML repository, import/export the data, start analysis tools and workflows, and provides detailed access to complex BioUML objects (e.g. models). In contrast with R scripts, JavaScript is executed inside the BioUML server.
- **Integration with Galaxy**. The Galaxy platform provides explicit descriptions (Galaxy tool XML file) of parameters for thousands of biological tools, mainly command line tools. BioUML extends the Galaxy tool configuration syntax that allows a closer interaction between the Galaxy and BioUML systems (http://wiki.biouml.org/index.php/Creating_Galaxy_tool).
- BioUML can read these XML files and generate forms where the user can specify values for corresponding parameters of the tools integrated in Galaxy.
- **Workflows**. For reproducible research, analysis tools can be joined into workflows. BioUML provides a powerful editor to visually construct workflows, and the engine for workflow execution is located on a server or cloud.

BioUML workflows can include the following component types:

- Analysis method: Method for analyses with specified inputs/outputs and parameters. It can be a BioUML method, Galaxy tool or Java wrapper for R functions.
- Analysis script: R script or JavaScript code, R methods.
- Analyses parameter: Subset of parameters that the user should specify to start the workflow.
- Analyses expression: Used to set and connect the input and output analysis parameters in the workflow.
- Cycle: Subset of workflow steps that will execute repeatedly. Cycles can iterate over the elements of folder, over table columns, over ranges of integers and over arrays of elements. See http://wiki.biouml.org/index.php/Workflow for more details.

## PATHWAY VISUALIZATION

The BioUML diagram editor/viewer can be used not only for visual modelling but also for the visualization of different biological pathways. For this purpose, the BioUML server contains the following databases: Reactome (37), PantherDB (38) and Biomodels (https://www.ebi.ac.uk/biomodels/). One can load their own pathways in the following formats: BioPAX, Antimony, SBGN-ML, SBML and Cytoscape CX (39).

BioUML utilizes several algorithms for the automatic layout of visual diagrams, including Hierarchical, Force-directed, Greedy and Grid layouts (40).

Data from omics experiments (transcriptomics, proteomics, metabolomics) can be mapped for different biological pathways and visualized by highlighting corresponding nodes on the diagram (http://wiki.biouml.org/index.php/Expression_mapping).

### Integrated genome browser

BioUML provides a fully integrated genome browser (41) that supports most of the features available in other modern genome browsers and comprises a comprehensive set of visualization tools for data processing results, which is extensively used to visualize information from a GTRD database (42).

### Collaborative reproducible research

User data (tables, diagrams, etc.) in BioUML are organized into projects. The administrator (creator) of the project can invite other users to participate in the project and manage their permissions. The user registration and management of access rights are performed via a central authentication and authorization system (https://bio-store.org). All user actions in a project, including performed analyses and scripts, are tracked in the project journal.

BioUML provides a collaborative editing functionality. Numerical models, pathways and workflows can be simultaneously modified by several researchers, and changes are instantly reflected on the screens of all users, while an embedded chat function facilitates user coordination and collaboration. The system also supports revision control and the possibility to revert to previous versions.

## USE CASES

### From virtual cell to virtual patient

The BioUML vision is to provide a computational platform to build virtual cells, virtual physiological humans and virtual patients. We have created two databases on the BioUML server that demonstrate our work in this direction using the BioUML platform.

The **Virtual cell** database includes three projects:

- The modular model of apoptosis (23) is the most detailed modern model of apoptosis. The model is split into 13 modules that comprise 280 species (proteins, their complexes, modifications such as different forms of the same molecule, and transformations e.g. phosphorylation) and 372 reactions, applying mass action, as well as Michaelis–Menten kinetics, with 459 parameters.
- CD95 and NF-κB signalling pathways (43): When identifying parameters on the basis of experimental data for human cell lines, we were faced with the problem of model overfitting. To solve this problem, we used the technique of model reduction, which allowed us to obtain a valid set of parameters supported by a sufficient amount of experimental data for modules related to the CD95 and NF-κB signalling pathways.
- Complex model of *Mycoplasma genitalium* cell (44): The model consists of 28 submodels utilizing different mathematical formalisms (ODE, stochastic, FBA). Originally, this model was implemented in MATLAB. In 2016, several research groups tried to recreate this model using SBML and SBGN standards (45). Only two submodels were completely finished—Cytokinesis and FtsZ Polymerization.

The **Virtual human** database includes a number of modular models that describe human physiology, including a classic model of blood circulation (46), a model of heart pumping and blood flow (47), a comprehensive model of blood flow through 55 of the largest arteries in the human body (48) and models with a focus on the regulation of blood volume (including kidney) (49,50).

**Antihypertensive drugs**: This is a database of pharmacokinetic (PK) and pharmacodynamic (PD) models of antihypertensive drugs from different drug groups, including aliskiren, losartan, amlodipine, enalapril, bisoprolol and hydrochlorothiazide.

**Complex model**: This database combines physiological models with PK/PD models to build so-called ‘virtual patients’. These can be created in diverse forms using different parts of human physiological models, with different focuses on subsystems depending on the research objectives.

**Virtual muscle** (51): This is a detailed kinetic model describing both the facilitated and passive transport of metabolites between muscle tissues and blood vessels and metabolic processes in cellular compartments (cytosol and mitochondria). We have rebuilt this model as a modular model that became an example of a multilevel model, taking into account cellular compartments and tissue organization.

### GTRD database

The GTRD database demonstrates how the BioUML platform can be used to a build web interface for access to a database. We have developed a special GTRD perspective (42,52) that provides browsing, information display, advanced search possibilities, and integration of the genome browser and information from the Ensembl database (gene structures, repeats, etc.) to visualize the GTRD data.

### Workflows as a cookbook for the analysis of omics data

Each workflow can be considered as a ready recipe for the specific analysis of corresponding omics data. A scientist needs only to import data, select the appropriate recipe, specify input/output data and press the ‘Run’ button. The platform will automatically analyse the data. This was a key idea of a geneXplain platform (http://genexplain.com/genexplain-platform/) (53) that now provides hundreds of workflows for the analysis of different types of omics data (microarrays, transcriptomics, proteomics, metabolomics, etc.). The geneXpain platform is a branch of the BioUML tree, with the focus on commercial application. It includes such commercial databases as TRANSFAC^®^ (transcription factors and their binding sites in a genome; 54), TRANSPATH^®^ (signal transduction network in eukaryotic cells; 55) and HumanPSD^®^ (disease biomarkers, drugs and clinical trials; 56). The geneXplain platform contains several of its own sophisticated methods for promoter and pathway analysis, such as Match™ (57) for the identification of transcription factor binding sites, CMA (Composite Module Analysis; 58) for the identification of composite regulatory modules in promoters and enhancers, tools for finding master regulators (59) in networks and other tools.

Recently, a new tool, Genome Enhancer (http://my-genome-enhancer.com/), has been developed based on the BioUML platform. Genome Enhancer is a tool for the fully automated analysis of multi-omics data. Depending on a user’s data, the platform automatically generates a corresponding workflow, executes the full analysis and presents the results as a well-structured detailed research article.

## DISCUSSION

The BioUML platform spans a comprehensive range of capabilities, including access to biological databases, powerful tools for systems biology (visual modelling, simulation, parameters fitting and analyses), a genome browser, scripts (R, JavaScript) and workflows for a diverse array of biomedical data analysis. There is a range of other software platforms that provide similar capabilities for data analysis and modelling with specific extensions for systems biology. The most prominent are:

- **R Studio** provides a web interface and R/Bioconductor provides hundreds of packages for biomedical data analysis.
- **MATLAB** has several packages for biomedical data analysis and systems biology, including SimBiology and IQM Tools (https://iqmtools.intiquan.com/, formerly Systems Biology Toolbox). SimuLink provides a powerful tool for the visual development of modular models. For instance, the comprehensive complex model of the bacterial cell, *Mycoplasma genitalium* (44) was created using MATLAB.
- **Jupyter notebook** (60) is widely used interactive computing environment across dozens of programming languages (Python, R, Julia and Scala).

Comprehensive comparisons of the BioUML platform with the above platforms, as well as comparisons with specialized tools for systems biology (e.g. CellDesigner (61), Tellurium (62), COPASI (63), iBioSim (64)), pathway visualization and analyses (e.g. Cytoscape (39)) and workflow platforms (Galaxy, Taverna (65)) are available at http://wiki.biouml.org/index.php/Tools_Comparison.

In general, the BioUML platform has the following advantages:

- A state-of-the-art simulation engine that supports visual modelling using different approaches. As mentioned above, BioUML is the only platform that can pass the SBML semantic test suite, including hierarchical models.
- It provides capabilities for both steps of the systems biology ‘dry-wet-dry’ cycle—the modelling and analysis of omics data.
- The platform can provide a perspective mechanism to facilitate a user’s focus on the tasks and databases they are working with.

## References

[1] Kolpakov F.A. BioUML – framework for visual modeling and simulation of biological systems. Proc. Int. Conf. Bioinf. Genome Regul. Struct. (BGRS'2002), Novosibirsk. 2002; 2:128–131.

[2] Kolpakov F.A., Ananko E.A., Kolesov G.B., Kolchanov N.A. GeneNet: a gene network database and its automated visualization. Bioinformatics. 1998; 14:529–537. 9694992 10.1093/bioinformatics/14.6.529

[3] Kolpakov F.A., Ananko E.A. Interactive data input into the GeneNet database. Bioinformatics. 1999; 15:713–714. 10487877 10.1093/bioinformatics/15.7.713

[4] Kolpakov F., Sharipov R., Cheremushkina E., Kalashnikova E. Biopath – a new approach to formalized description and simulation of biological systems. Proc. Int. Conf. Bioinf. Genome Regul. Struct. (BGRS’2006), Novosibirsk. 2006; 3:96–100.

[5] Kolpakov F., Poroikov V., Sharipov R., Kondrakhin Y., Zakharov A., Lagunin A., Milanesi L., Kel A. CYCLONET - an integrated database on cell cycle regulation and carcinogenesis. Nucleic Acids Res. 2007; 35:D550–D556. 17202170 10.1093/nar/gkl912 PMC1899094

[6] Le Novère N., Hucka M., Mi H., Moodie S., Schreiber F., Sorokin A., Demir E., Wegner K., Aladjem M.I., Wimalaratne S.M. et al. The Systems biology graphical notation. Nature Biotechnol. 2009; 27:735–741. 19668183 10.1038/nbt.1558

[7] Shampine L.F., Reichelt M.W. The MATLAB ODE Suite. SIAM J. Sci. Comput. 1997; 18:1–22.

[8] Hucka M., Smith L., Bergmann F., Keating S.M. SBML Test Suite release 3.3.0. 2017; https://zenodo.org/record/1112521#.XNzsIJwxXmE.

[9] Kholodenko B.N., Sauro H.M. Alberghina L, Westerhoff HV Mechanistic and modular approaches to modeling and inference of cellular regulatory networks. Systems Biology: Definitions and Perspectives. Topics in Current Genetics. 2005; 13:Berlin Springer-Verlag 357–451.

[10] Ramos M., Schiffer L., Re A., Azhar R., Basunia A., Rodriguez C., Chan T., Chapman P., Davis S.R., Gomez-Cabrero D. et al. Software for the integration of multiomics experiments in bioconductor. Cancer Res. 2017; 77:e39–e42. 29092936 10.1158/0008-5472.CAN-17-0344 PMC5679241

[11] Afgan E., Baker D., Batut B., van den Beek M., Bouvier D., Cech M., Chilton J., Clements D., Coraor N., Grüning B.A. et al. The Galaxy platform for accessible, reproducible and collaborative biomedical analyses: 2018 update. Nucleic Acids Res. 2018; 46:W537–W544. 29790989 10.1093/nar/gky379 PMC6030816

[12] Demir E., Cary M.P., Paley S., Fukuda K., Lemer C., Vastrik I., Wu G., D'Eustachio P., Schaefer C., Luciano J. et al. The BioPAX community standard for pathway data sharing. Nat. Biotechnol. 2010; 28:935–942. 20829833 10.1038/nbt.1666 PMC3001121

[13] Hucka M., Finney A., Sauro H.M., Bolouri H., Doyle J.C., Kitano H., Arkin A.P., Bornstein B.J., Bray D., Cornish-Bowden A. et al. The Systems Biology Markup Language (SBML): A medium for representation and exchange of biochemical network models. Bioinformatics. 2003; 19:524–531. 12611808 10.1093/bioinformatics/btg015

[14] Olivier B., Bergmann F. SBML level 3 package: flux balance constraints version 2. J. Integr. Bioinform. 2018; 15:20170082. 10.1515/jib-2017-0082 PMC6167036 29522419

[15] Smith L.P., Hucka M., Hoops S., Finney A., Ginkel M., Myers C.J., Moraru I., Liebermeister W. SBML Level 3 package: hierarchical model composition, version 1 release 3. J. Integr. Bioinform. 2015; 12:268. 26528566 10.2390/biecoll-jib-2015-268 PMC5451323

[16] Smith L.P., Bergmann F.T., Chandran D., Sauro M.H. Antimony: a modular model definition language. Bioinformatics. 2009; 25:2452–2454. 19578039 10.1093/bioinformatics/btp401 PMC2735663

[17] Waltemath D., Adams R., Bergmann F.T., Hucka M., Kolpakov F., Miller A.K., Moraru I.I., Nickerson D., Sahle S., Snoep J.L. et al. Reproducible computational biology experiments with SED-ML–The Simulation Experiment Description Markup Language. BMC Syst Biol. 2011; 5:198. 22172142 10.1186/1752-0509-5-198 PMC3292844

[18] Hindmarsh A.C., Brown P.N., Grant K.E., Lee S.L., Serban R., Shumaker D.E., Woodward C.S. SUNDIALS: suite of nonlinear and differential/algebraic equation solvers. ACM Trans. Math. Soft. 2005; 31:363–396.

[19] Hairer E., Wanner G. Solving Ordinary Differential Equations II. Stiff and Differential-Algebraic Problems. Springer Series in Computational Mathematics 14. 1996; Second Edition Berlin, Heidelberg Springer-Verlag.

[20] Dormand J.R., Prince P.J. A family of embedded Runge-Kutta formulae. J. Comput. Appl. Math. 1980; 6:19–26.

[21] Gillespie D.T. Stochastic simulation of chemical kinetics. Annu. Rev. Phys. Chem. 2007; 58:35–55. 17037977 10.1146/annurev.physchem.58.032806.104637

[22] Gibson M.A., Bruck J. Efficient exact stochastic simulation of chemical systems with many species and many channels. J. Phys. Chem. A. 2000; 104:1876–1889.

[23] Kutumova E.O., Kiselev I.N., Sharipov R.N., Lavrik I.N., Kolpakov F.A. A modular model of the apoptosis machinery. Adv. Experim. Med. Biol. 2012; 736:235–245. 10.1007/978-1-4419-7210-1_13 22161332

[24] Kiselev I.N., Semisalov B.V., Biberdorf E.A., Sharipov R.N., Blokhin A.M., Kolpakov F.A. Modular modeling of the human cardiovascular system. Math. Biol. Bioinform. 2012; 7:703–736.

[25] Kutumova E., Ryabova A., Valeev T., Kolpakov F. BioUML plug-in for nonlinear parameter estimation using multiple experimental data. Virt. Biol. 2013; 1:47–58.

[26] Runarsson T.P., Yao X. Stochastic ranking for constrained evolutionary optimization. IEEE Trans. Evol. Comput. 2000; 4:284–294.

[27] Sierra M.R., Coello Coello C.A. Coello CA, Hernández Aguirre A, Zitzler E Improving pso-based multi-objective optimization using crowding, mutation and ∈-dominance. Evolutionary Multi-Criterion Optimization. EMO 2005. Lecture Notes in Computer Science. 2005; 3410:Berlin, Heidelberg Springer 505–519.

[28] Nebro A.J., Durillo J.J., Luna F., Dorronsoro B., Alba E. MOCell: A cellular genetic algorithm for multiobjective optimization. Int. J. Intell. Syst. 2009; 24:726–746.

[29] Raue A., Kreutz C., Maiwald T., Bachmann J., Schilling M., Klingmüller U., Timmer J. Structural and practical identifiability analysis of partially observed dynamical models by exploiting the profile likelihood. Bioinformatics. 2009; 25:1923–1929. 19505944 10.1093/bioinformatics/btp358

[30] Raue A., Becker V., Klingmüller U., Timmer J. Identifiability and observability analysis for experimental design in nonlinear dynamical models. Chaos. 2010; 20:045105. 21198117 10.1063/1.3528102

[31] Gorban A.N., Radulescu O., Zinovyev A.Y. Asymptotology of chemical reaction networks. Chem. Engineer. Sci. 2009; 65:2310–2324.

[32] Choi J., Yang K.W., Lee T.Y., Lee S.Y. New time-scale criteria for model simplification of bio-reaction systems. BMC Bioinform. 2008; 9:338. 10.1186/1471-2105-9-338 PMC2553091 18694523

[33] Rabitz H., Kramer M., Dacol D. Sensitivity analysis in chemical kinetics. Ann. Rev. Phys. Chem. 1983; 34:419–461.

[34] Reder C. Metabolic control theory: a structural approach. J. Theor. Biol. 1988; 135:175–201. 3267767 10.1016/s0022-5193(88)80073-0

[35] Sauro H.M., Ingalls B. Conservation analysis in biochemical networks: computational issues for software writers. Biophys. Chem. 2004; 109:1–15. 15059656 10.1016/j.bpc.2003.08.009

[36] Huber W., Carey V.J., Gentleman R., Anders S., Carlson M., Carvalho B.S., Bravo H.C., Davis S., Gatto L., Girke T. et al. Orchestrating high-throughput genomic analysis with Bioconductor. Nat. Methods. 2015; 12:115–121. 25633503 10.1038/nmeth.3252 PMC4509590

[37] Croft D., O’Kelly G., Wu G., Haw R., Gillespie M., Matthews L., Caudy M., Garapati P., Gopinath G., Jassal B. et al. Reactome: A database of reactions, pathways and biological processes. Nucleic Acids Res. 2011; 39:D691–D697. 21067998 10.1093/nar/gkq1018 PMC3013646

[38] Thomas P.D., Kejariwal A., Campbell M.J., Mi H., Diemer K., Guo N., Ladunga I., Ulitsky-Lazareva B., Muruganujan A., Rabkin S. et al. PANTHER: a browsable database of gene products organized by biological function, using curated protein family and subfamily classification. Nucleic Acids Res. 2003; 31:334–341. 12520017 10.1093/nar/gkg115 PMC165562

[39] Shannon P., Markiel A., Ozier O., Baliga N.S., Wang J.T., Ramage D., Amin N., Schwikowski B., Ideker T. Cytoscape: a software environment for integrated models of biomolecular interaction networks. Genome Res. 2003; 13:2498–2504. 14597658 10.1101/gr.1239303 PMC403769

[40] Kaname K., Masao N., Satoru M. Fast grid layout algorithm for biological networks with sweep calculation. Bioinformatics. 2008; 24:1433–1441. 18424458 10.1093/bioinformatics/btn196

[41] Valeev T., Yevshin I., Kolpakov F. BioUML genome browser. Virt. Biol. 2013; 1:15–26.

[42] Yevshin I., Sharipov R., Kolmykov S., Kondrakhin Y., Kolpakov F. GTRD: a database on gene transcription regulation-2019 update. Nucleic Acids Res. 2019; 47:D100–D105. 30445619 10.1093/nar/gky1128 PMC6323985

[43] Kutumova E., Zinovyev A., Sharipov R., Kolpakov F. Model composition through model reduction: a combined model of CD95 and NF-κB signaling pathways. BMC Syst. Biol. 2013; 7:13. 23409788 10.1186/1752-0509-7-13 PMC3626841

[44] Karr J.R., Sanghvi J.C., Macklin D.N., Assad-Garcia N., Glass J.I. A whole-cell computational model predicts phenotype from genotype. Cell. 2012; 150:389–401. 22817898 10.1016/j.cell.2012.05.044 PMC3413483

[45] Waltemath D., Karr J.R., Bergmann F.T., Chelliah V., Hucka M. Toward community standards and software for whole-cell modeling. IEEE Trans. Biomed. Engineer. 2016; 63:2007–2014. 10.1109/TBME.2016.2560762 PMC5451320 27305665

[46] Guyton A.C., Coleman T.G., Granger H.J. Circulation: overall regulation. Ann. Rev. Physiol. 1972; 34:13–46. 4334846 10.1146/annurev.ph.34.030172.000305

[47] Proshin A.P., Solodyannikov Y. Identification of the parameters of blood circulation system. Automat. Remote Control. 2010; 71:1629–1637.

[48] Biberdorf E.A., Blokhin A.M., Trakhinin Y.L. Ivanova AL, Markel AM, Blokhin EV, Mishchenko Global modeling of the human arterial system. Circulatory System and Arterial Hypertension: Experimental Investigation, Mathematical and Computer Simulation. 2012; NY Nova Science Publishers, Inc 115–142.

[49] Karaaslan F., Denizhan Y., Kayserilioglu A., Ozcan Gulcur H. Long-term mathematical model involving renal sympathetic nerve activity, arterial pressure, and sodium excretion. Annals Biomed. Engineer. 2005; 33:1607–1630. 10.1007/s10439-005-5976-4 16341927

[50] Hallow K.M., Lo A., Beh J., Rodrigo M., Ermakov S., Friedman S., de Leon H., Sarkar A., Xiong Y., Sarangapani R. et al. A model-based approach to investigating the pathophysiological mechanisms of hypertension and response to antihypertensive therapies: Extending the Guyton model. Am. J. Physiol. Reg. Integr. Comp. Physiol. 2014; 306:R647–R662. 10.1152/ajpregu.00039.2013 24500431

[51] Li Y., Dash R.K., Kim J., Saidel G.M., Cabrera M.E. Role of NADH/NAD+ transport activity and glycogen store on skeletal muscle energy metabolism during exercise: *in silico* studies. Am. J. Physiol. Cell Physiol. 2009; 296:25–46. 10.1152/ajpcell.00094.2008 PMC2636997 18829894

[52] Yevshin I., Sharipov R., Valeev T., Kel A., Kolpakov F. GTRD: a database of transcription factor binding sites identified by ChIP-seq experiments. Nucleic Acids Res. 2017; 45:D61–D67. 27924024 10.1093/nar/gkw951 PMC5210645

[53] Kolpakov F., Poroikov V., Selivanova G., Kel A. GeneXplain — identification of causal biomarkers and drug targets in personalized cancer pathways. J. Biomol. Tech. 2011; 22:S16.

[54] Wingender E. The TRANSFAC project as an example of framework technology that supports the analysis of genomic regulation. Brief. Bioinformatics. 2008; 9:326–332. 18436575 10.1093/bib/bbn016

[55] Krull M., Voss N., Choi C., Pistor S., Potapov A., Wingender E. TRANSPATH: an integrated database on signal transduction and a tool for array analysis. Nucleic Acids Res. 2003; 31:97–100. 12519957 10.1093/nar/gkg089 PMC165536

[56] Michael H., Hogan J., Kel A., Kel-Margoulis O., Schacherer F., Voss N., Wingender E. Building a knowledge base for systems pathology. Brief. Bioinformatics. 2008; 9:518–531. 19073714 10.1093/bib/bbn038

[57] Kel A.E., Gössling E., Reuterm I., Cheremushkin E., Kel-Margoulis O.V., Wingender E. MATCH: A tool for searching transcription factor binding sites in DNA sequences. Nucleic Acids Res. 2003; 31:3576–3579. 12824369 10.1093/nar/gkg585 PMC169193

[58] Weber A., Dittrich-Breiholz O., Schneider H., Kel A., Jauregui R., Wingender E., Kracht M. Identification of composite promoter modules in inflammation-regulated genes. Cell. Commun. Signal. 2009; 7:A105.

[59] Kel A.E., Stegmaier P., Valeev T., Koschmann J., Poroikov V., Kel-Margoulis O.V., Wingender E. Multi-omics “upstream analysis” of regulatory genomic regions helps identifying targets against methotrexate resistance of colon cancer. EuPA Open Proteom. 2016; 13:1–13. 29900117 10.1016/j.euprot.2016.09.002 PMC5988513

[60] Perkel J.M. Why Jupyter is data scientists' computational notebook of choice. Nature. 2018; 563:145–146. 30375502 10.1038/d41586-018-07196-1

[61] Funahashi A., Matsuoka Y., Jouraku A., Morohashi M., Kikuchi N., Kitano H. CellDesigner 3.5: a versatile modeling tool for biochemical networks. Proc. IEEE. 2008; 96:1254–1265.

[62] Choi K., Medley J.K., König M., Stocking K., Smith L., Gu S., Sauro H.M. Tellurium: an extensible python-based modeling environment for systems and synthetic biology. Biosystems. 2018; 171:74–79. 30053414 10.1016/j.biosystems.2018.07.006 PMC6108935

[63] Hoops S., Sahle S., Gauges R., Lee C., Pahle J., Simus N., Singhal M., Xu L., Mendes P., Kummer U. COPASI: a complex pathway simulator. Bioinformatics. 2006; 22:3067–3074. 17032683 10.1093/bioinformatics/btl485

[64] Watanabe L., Nguyen T., Zhang M., Zundel Z., Zhang Z., Madsen C., Roehner N., Myers C. iBioSim 3: a tool for model-based genetic circuit design. ACS Synth. Biol. 2018; doi:10.1021/acssynbio.8b00078. 10.1021/acssynbio.8b00078 29944839

[65] Wolstencroft K., Haines E., Fellows D., Williams A., Withers D., Owen S., Soiland-Reyes S., Dunlop I., Nenadic A., Fisher P. et al. The Taverna workflow suite: designing and executing workflows of Web Services on the desktop, web or in the cloud. Nucleic Acids Res. 2013; 41:W557–W561. 23640334 10.1093/nar/gkt328 PMC3692062

